# Neuromyelitis optica spectrum disorders: a review with a focus on children and adolescents

**DOI:** 10.1055/s-0043-1761432

**Published:** 2023-03-22

**Authors:** Renata Barbosa Paolilo, José Albino da Paz, Samira Luisa Apóstolos-Pereira, Carolina de Medeiros Rimkus, Dagoberto Callegaro, Douglas Kazutoshi Sato

**Affiliations:** 1 Universidade de São Paulo, Faculdade de Medicina, Hospital das Clínicas, Departamento de Neurologia, São Paulo SP, Brazil.; 2 Universidade de São Paulo, Faculdade de Medicina, Hospital das Clínicas, Departamento de Radiologia, São Paulo SP, Brazil.; 3 Pontifícia Universidade Católica do Rio Grande do Sul, Instituto do Cérebro do Rio Grande do Sul, Porto Alegre RS, Brazil.

**Keywords:** Aquaporin 4, Multiple Sclerosis, Myelin-Oligodendrocyte Glycoprotein, Myelitis, Optic Neuritis, Neuromyelitis Optica, Aquaporina 4, Esclerose Múltipla, Glicoproteína Mielina-Oligodendrócito, Mielite, Neurite Óptica, Neuromielite Óptica

## Abstract

Neuromyelitis optica spectrum disorder (NMOSD) is a rare and severe inflammatory disorder of the central nervous system (CNS). It is strongly associated with anti-aquaporin 4 antibodies (AQP4-IgG), and it mainly affects young women from non-white ethnicities. However, ∼ 5 to 10% of all cases have onset during childhood. Children and adolescents share the same clinical, radiologic, and laboratory presentation as adults. Thus, the same NMOSD diagnostic criteria are also applied to pediatric-onset patients, but data on NMOSD in this population is still scarce. In seronegative pediatric patients, there is a high frequency of the antibody against myelin oligodendrocyte glycoprotein (MOG-IgG) indicating another disease group, but the clinical distinction between these two diseases may be challenging. Three drugs (eculizumab, satralizumab, and inebilizumab) have been recently approved for the treatment of adult patients with AQP4-IgG-positive NMOSD. Only satralizumab has recruited adolescents in one of the two pivotal clinical trials. Additional clinical trials in pediatric NMOSD are urgently required to evaluate the safety and efficacy of these drugs in this population.

## Resumo

O espectro da neuromielite óptica (ENMO) é uma rara e grave doença inflamatória do sistema nervoso central (SNC), fortemente associada ao anticorpo anti-aquaporina 4 (AQP4-IgG) e que afeta preferencialmente mulheres jovens de etnias não-caucasianas. No entanto, aproximadamente de 5 a 10% de todos os casos se iniciam na infância. Crianças e adolescentes compartilham as mesmas características clínicas, radiológicas e laboratoriais dos adultos. Além disso, o mesmo critério diagnóstico de ENMO é aplicado para pacientes com início na infância. No entanto, dados da população pediátrica são escassos. Em pacientes pediátricos soronegativos, existe uma alta frequência de positividade ao anticorpo contra a glicoproteína na mielina do oligodendrócito (MOG-IgG), indicando outra patologia; porém, a distinção clínica entre as duas doenças é desafiadora. Três medicações (eculizumabe, inebilizumabe e satralizumabe) foram recentemente aprovadas para pacientes adultos com AQP4-IgG. Apenas um dos ensaios pivotais do satralizumabe recrutou adolescentes. Novos ensaios clínicos em pacientes pediátricos com ENMO são necessários para avaliar a segurança e eficácia destas drogas nesta população.

## INTRODUCTION

Neuromyelitis optica (NMO) is an autoimmune demyelinating inflammatory disease of the central nervous system (CNS). 1 Described by Devic and Gault in 1894, NMO was considered a variant of Multiple Sclerosis (MS) for many years. 2

In 2004, the discovery of the aquaporin 4 antibody (AQP4-IgG) and the evidence of its pathogenicity changed the pathophysiological understanding of the disease, definitively differentiating it from MS. ^3^ With the identification of a more significant number of positive anti-AQP4 cases, it was also possible to expand the spectrum of NMO including atypical and incomplete forms of the disease, introducing the concept of neuromyelitis optica spectrum disorders (NMOSD). 3 More recently, studies showed that some AQP4-IgG seronegative patients were positive for the antibody against myelin oligodendrocyte glycoprotein (MOG-IgG). In 2015, the diagnostic criteria for NMOSD were revised and stratified by the AQP4-IgG positivity. 1

Neuromyelitis optica spectrum disorder is a rare disease distributed worldwide. It affects preferably adults and females, with a gender ratio of up to 9:1. 4 Approximately 5 to 10% of NMOSD cases start before 18 years old. 5 Pediatric-onset NMOSD patients have similar manifestations as adults, and the new diagnostic criteria also apply to this age group. 1 5 Due to the low number and heterogeneity of published studies, data on NMOSD in childhood and adolescents are scarce, especially regarding treatment and clinical outcome. 6 7

The present paper provides a comprehensive review of pediatric NMOSD focusing on historical aspects, disease pathophysiology, epidemiology, clinical and radiological aspects, treatment recommendations, and, ultimately, a summary of the published pediatric cohorts.

## HISTORICAL ASPECTS

**Figure 1:**
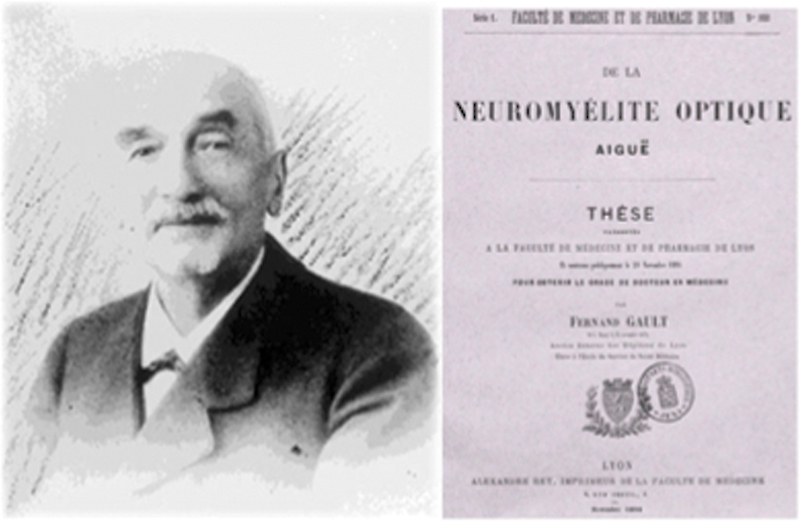
Eugène Devic (left) and his pupil Fernand Gault doctoral thesis (right) Adapted from Jarius et al., 2013. 8

Although the expression “Acute Neuromyelitis” was first used by Eugène Devic in 1894 in a Medical Conference in Lyon, several case reports on patients with acute myelitis and optic neuritis (ON) have been reported before in the literature. 8 The French physician Antoine Portal was probably the first to identify a case in 1804. 9 Devic described the case series of 17 patients with acute ON and myelitis. In November of the same year, Fernand Gault published his doctoral thesis with a detailed clinical-pathological analysis on the Devic patient's case. ( Figure 1 ) The term “Devic Disease” was suggested in 1907 by Peppo Acchioté and was used in reference to Devic's description of a monophasic disease affecting the optic nerve and spinal cord. 8

In the middle of the 20 ^th^ century, > 300 cases of NMO had been reported. A better understanding of the disorder permitted the expansion of the disease manifestation beyond Devic's description. 10 It was recognized that ON could be unilateral or bilateral and the occurrence of ON and acute myelitis may have an interval from weeks to years. 2 Nevertheless, there was some limitation to differentiate NMO from MS. In Asia, for example, the term Optic-spinal Multiple Sclerosis was used to classify the patients with severe ON or acute myelitis without brain or cerebellar involvement. 10

The revision of 71 patients from the Mayo Clinic allowed a group of investigators to propose the first NMO diagnostic criteria. Compared with MS, the NMO cases presented with a more severe presentation, cerebral spinal fluid (CSF) cell count > 50 cell/mm3, normal brain magnetic resonance imaging (MRI), and extensive spinal cord lesions involving more than three vertebral segments. 11

In 2004, the same group reported the presence of an IgG antibody in the serum of NMO and Optic-spinal MS patients, suggesting that they were actually the same disease. The high specificity of the antibody also allowed the distinction from MS. ^3^ Subsequently, they reported that the antibody was selective to the aquaporin-4 water channel located in the endfeet of astrocytes. 12

Due to the high specificity of the antibody, the diagnostic criteria were updated in 2006 to include AQP4-IgG testing. 13 Moreover, there was an increased recognition of AQP4-IgG patients with limited disease manifestations, including patients with monophasic ON, acute myelitis, or patients with other systemic disorders. Brain involvement has also been identified in many NMOSD patients, mainly in regions expressing high-levels of AQP4, such as periependymal regions. 1 3

At the same time, there were advances in the role of MOG-IgG in inflammatory demyelinating CNS disorders. Different from studies from the early 2000s, new studies found that MOG-IgG was associated with a non-MS phenotype. 14 15 Interestingly, many MOG-IgG-positive patients had clinical manifestations similar to AQP4-IgG NMOSD. This antibody can be found in up to 30% of NMO patients who tested negative to AQP4-IgG. 16 However, the clinical and MRI findings of MOG-associated disease (MOGAD) goes beyond the NMOSD phenotype, expanding the new concept of MOGAD. 17

During the decade of 2010, studies concluded that MS modifying therapies were ineffective in preventing NMO attacks and could also harm patients' clinical status. This information is essential to guide the correct NMO diagnosis and treatment.

In 2015, the International Panel for NMO Diagnosis (IPND) updated the diagnostic criteria. The IPND recommended the term NMOSD to unify all cases of NMO, Optical-spinal MS, and Devic Disease. The new criteria stratified patients according to the AQP4-IgG positivity, relying on six clinical core features and MRI findings 1 ( Table 1 ).

**Table 1:** Diagnostic criteria for Neuromyelitis optica spectrum disorders – International Panel for NMO diagnosis 2015

**Positive AQP4-IgG**	1. At least one clinical syndrome 2. Positive AQP4-IgG by the best assay 3. Exclusion of alternative diagnosis
**Negative or unknown AQP4-IgG**	1. At least two clinical syndromes - at least one must be: Optic Neuritis, LETM or area postrema syndrome - evidence of dissemination in space - fulfill additional MRI criteria 2. Negative AQP4-IgG by the best assay or unavailable antibody testing 3. Exclusion of alternative diagnosis
**Clinical syndromes**	1. Optic neuritis 2. Acute myelitis 3. Area postrema syndrome 4. Brainstem syndrome 5. Diencephalic syndrome 6. Cerebral syndrome
**Additional MRI criteria**	1. Acute optic neuritis - normal brain MRI or unspecific findings, or - optic nerve MRI with T2-hyperintensive lesion or gadolinium enhancement extending over > ½ optic nerve length or involving optic chiasm 2. Acute myelitis -intramedullary lesion extending over ≥ 3 contiguous segments (LETM), or -spinal cord atrophy ≥ 3 segments in a patient with history compatible with acute myelitis 3. Area postrema syndrome: dorsal medula/area postrema lesions 4. Acute brainstem syndrome: periependymal brainstem lesions

Abbreviations: AQP4-IgG, aquaporin 4 antibody; LETM, longitudinally extensive transverse myelitis; MRI, magnetic resonance imaging.

For the last 3 years, the most remarkable advances for NMOSD were the publication of positive results from four different clinical trials evaluating the efficacy and safety of three different drugs, 18 19 20 21 resulting in the approval of these treatments by international agencies. 22 At this time, only satralizumab is licensed in Brazil.

Currently, NMOSD research focuses on identifying disease biomarkers that could predict attacks, prognosis, and guide the best therapeutic choices. 23

## AQUAPORIN-4 AND NMOSD PATHOPHYSIOLOGY

Aquaporin-4 is the most abundant water channel of the CNS, being expressed predominantly in the astrocyte endfeet of the blood-brain barrier regulating water influx in synapses, injuries, and the process of cellular migration. 2 It is also expressed in other organs such as kidneys, skeletal muscles, and parietal cells, but pathological effects of AQP4-IgG in these organs are not seen in most of the patients due to more effective cellular protection mechanisms, such as CD59 expression inhibiting the complement complex formation. 2 24

In humans, there are 2 relevant aquaporin-4 isoforms that can be differentiated if the transcription at the N-terminal starts at the 1 ^st^ (M1) of 23 ^rd^ (M23) methionine. 22 The monomers M1 and M23 are composed of eight helical segments associated with the membrane, with a pore for selective passage of water by an osmotic gradient. The organization of monomers into tetrameters and, in sequence, into orthogonal arrangements seems to facilitate the cognition of aquaporin-4 by the AQP4-IgG. 25 26

The AQP4-IgG is an antibody from the G1 subclass, thus a competent complement activator. It is mainly produced in the periphery and enters the CNS through the blood-brain barrier, the capillaries of the circumventricular organs, the meninges, or the parenchymal blood vessels. 22 Antibody binding causes its internalization or reorganization and aggregation, complement deposition in the foot of astrocytes, and inflammatory infiltrate rich in macrophages, neutrophils, eosinophils, and T and B lymphocytes. 2 22 26 The astrocytic loss is the main histological finding, evidenced by the loss of aquaporin-4 and the astrocytic marker glial fibrillary acidic protein (GFAP). 26 Depending on the severity of the tissue injury and the stage of the disease, it can be followed secondarily by the death of oligodendrocytes and neurons. 22

Animal and cellular studies have shown the AQP4-IgG pathogenicity. The intrathecal injection of serum containing AQP4-IgG and human complement in rodents resulted in NMO-like lesions with reduction of aquaporin-4, astrocytic loss, complement deposition, inflammatory infiltrate, demyelination, and necrosis. 27

Clinical studies suggested AQP4-IgG pathogenicity through different ways: high CSF AQP4-IgG titers and activated complement compounds during disease activity, high efficacy of treatments whose mechanisms of action reduced B cells and antibody levels, and the predilection of lesions in areas with high AQP4 expression. 3 22 Furthermore, other studies have indicated that antibody positivity in patients with ON or extensive myelitis was associated with recurrent NMOSD attacks. 28 29

## EPIDEMIOLOGY

The prevalence and incidence of NMOSD varies depending on the studied population and geographic area but are higher in black and Asian populations compared with Caucasians. 4 30

The Afro-Caribbean region has the highest annual incidence and prevalence globally of 0.73/100,000 and 10/100,000 people, respectively. On the other hand, Australia and New Zealand regions have the lowest incidence (0.037/100,000 people) and prevalence (0.7/100,000). 31 In the few epidemiological studies including pediatric patients, the annual incidence varied from 0.031 to 0.06/100,000 children and the prevalence varied from 0.06 to 0.22/100,000 children. 31

Neuromyelitis optica spectrum disorder is more common in the female sex, with a ratio varying from 3:1 to 9:1. The female prevalence is especially significant in patients seropositive to AQP4-IgG. In contrast, this ratio decreases to near 1:1 in patients with NMOSD and MOG-IgG. 32

The mean age of disease onset in AQP4-IgG positive patients is ∼ 40 years old. 32 There is a tendency for earlier onset in patients with non-white ethnicity. 33 Conversely, MOGAD is more prevalent in children. 17 34 Few studies reported the association of NMOSD with different alleles of the human leukocyte antigen (HLA) system, including HLA DRB1*03:01, DRB1*04:05, and DRB1*16:02. 35 36

The most important known risk factor for NMOSD is the female sex. Studies evaluating environmental factors such as vitamin D levels, dietary intake, and sleep patterns were inconclusive. 22 37 38 Several authors reported temporal association of the inaugural attack with different infections and, less commonly, vaccinations. 39 40 To date, there is no evidence of a clear association of NMOSD attacks with specific infections.

## CLINICAL, RADIOLOGIC AND LABORATORY ASPECTS

Attacks of ON and acute myelitis are frequently found in NMOSD. Other clinical syndromes were recognized as typical for NMOSD after the IPND-2015 diagnostic criteria: area postrema syndrome, brainstem syndrome, diencephalic syndrome, and cerebral syndrome. 1 3 4 The diagnosis is based on a characteristic clinical presentation supported by MRI findings. 1 41

**Figure 2:**
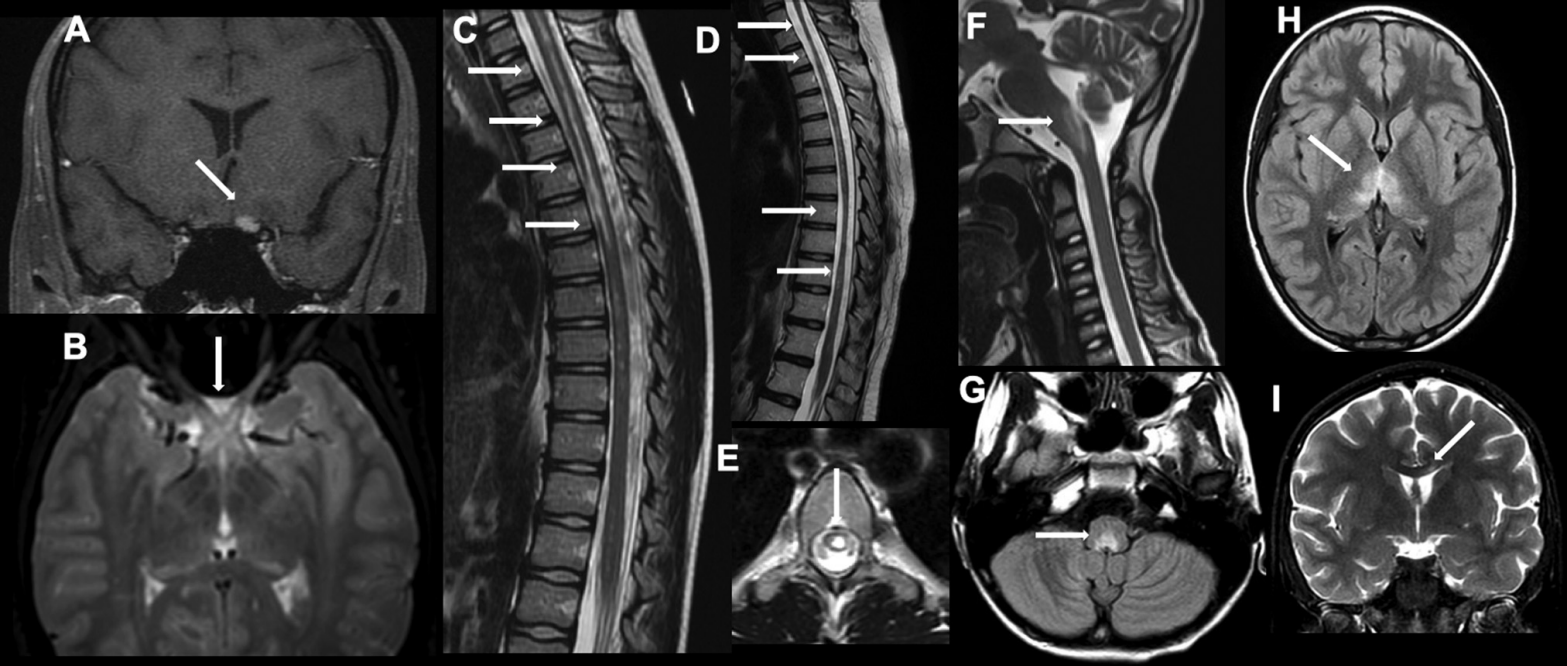
Typical Neuroimaging findings in pediatric AQP4-IgG positive neuromyelitis optica Spectrum Disorder. ( **A)** Unilateral optic neuritis; ( **B)** Bilateral optic neuritis with optic chiasm involvement; ( **C)** Longitudinally extensive transverse myelitis; ( **D)** Longitudinally extensive atrophy; ( **E)** Bright Spotty lesion; **(F)** and ( **G)** Area postrema involvement; ( **H)** Diencephalic lesion; ( **I)** Corpus callosum lesion.

Figure 2 depicts typical neuroimaging findings in pediatric-onset NMOSD patients.

For patients with positive AQP4-IgG, the ON typically presents with a severe acute or subacute unilateral or bilateral visual loss preceded by eye pain. The neurological examination may reveal low visual acuity, generally severe, color vision deficit, noncentral scotoma, and altitudinal defect. 22 The brain and orbital MRI may demonstrate T2 or FLAIR lesions or new lesions with gadolinium enhancement, generally extensive, involving more than half of the optic nerve, with a posterior predominance, frequently affecting the optic chiasm. 41 Optic neuritis associated with MOG-IgG resembles the AQP4-IgG presentation, but the bilateral and anterior involvement sparing the optic chiasma is more common. 22

Myelitis in NMOSD is generally longitudinally extensive transverse myelitis (LETM), involving more than three contiguous vertebral segments. Patients usually present with severe motor and sensory deficits, pain, and bladder symptoms. 22 In the acute phase, spinal cord MRI discloses an extensive, central, edematous lesion in T2/FLAIR, eventually with gadolinium enhancement. The upper cervical lesions may also extend to the medulla, promoting persistent hiccups, nausea and/or vomiting. Extensive atrophy can be found in chronic lesions. 42 Compared with patients with AQP4-IgG, myelitis in MOG-IgG patients presents with some similarities, but caudal segments of the spinal cord seem to be more frequently involved.

Area postrema syndrome occurs in up to 10 to15% of NMOSD patients positive for AQP4-IgG. Its clinical presentation is characterized by nausea, vomiting, and hiccups persisting for more than 72 hours (median of 14 days) and can result in weight loss. 43 Brain MRI may be normal or discloses lesions in the dorsal region of the medulla, especially the area postrema. Commonly, the lesion is bilateral and contiguous with the upper cervical lesion. 1

Brainstem syndrome can manifest clinically by ocular movement disorders, pruritus, hearing loss, facial palsy, trigeminal neuralgia, ataxia, and respiratory failure. 22 Brain MRI reveals periependymal lesions in the brainstem and cerebellum at T2/FLAIR.

Diencephalic syndrome is less frequent. Patients present with hypersomnia, narcolepsy, hypo or hyperthermia, syndrome of inappropriate antidiuretic hormone secretion, anorexia, obesity, anhidrosis, decreased level of consciousness. 44 Radiologic criteria include evidence of lesions involving the hypothalamus, thalamus or peripendymal region of the third ventricle. 1

Diffuse brain involvement is associated with encephalopathy, seizures, focal deficits, and hydrocephalus. The cerebral syndrome is less frequent in adults than in children and is more common in MOGAD than NMOSD associated with AQP4-IgG. Brain MRI discloses large edematous lesions involving the corpus callosum, the corticospinal tract, or periependymal regions. 1

For diagnosing NMOSD, it is crucial to exclude alternative diagnoses such as other demyelinating disorders, systemic inflammatory diseases, and active infections. 1

Brain, orbital, and spinal cord neuroimaging have an essential role in differentiating NMOSD from other disorders. Compared with NMOSD, MS patients have perpendicular lesions to the surface of the lateral ventricles, juxtacortical lesions involving the U or cortical fibers, and peripheral and short spinal cord lesions. 1 41 More recently, a hyperintense lesion on spinal MRI (bright spotty lesion) was recognized as a characteristic of AQP4-IgG NMOSD. 45

Besides neuroimaging, laboratory tests and CSF analysis can help in the NMOSD diagnosis. Lumbar puncture at the acute phase generally reveals slight to moderate cell count with a predominance of neutrophils and eosinophils, along with moderately elevated protein levels. Up to 25% of patients may test positive for oligoclonal bands (OCB), although this finding favors an MS diagnosis. 1

The coexistence of systemic autoimmune disorders is common in NMOSD patients, mainly systemic lupus erythematosus, Sjogren syndrome, and myasthenia gravis. 1 Positivity to serum unspecific autoimmune antibodies can be found in 38 to 45% of patients with NMOSD irrespective of AQP4-IgG positivity. 3 It is not clear if autoimmune coexistence implies patient's diagnosis or prognosis, but some treatments may help the management of both diseases. 46

According to the IPND-2015 diagnostic criteria, AQP4-IgG should be tested in the serum of patients presenting with clinical features typical for NMOSD, such as extensive myelitis, recurrent myelitis, area postrema syndrome, or severe ON. 1 22 28 There is no recommendation for testing AQP4-IgG in CSF on a clinical basis.

Assays for AQP4-IgG testing must have a high sensibility and specificity. 28 The first studies on NMOSD tested AQP4-IgG through tissue assays (immunofluorescence or ELISA), demonstrating 60% sensibility. Cell-based assays (CBAs) increased sensibility to 80% and specificity to almost 100%. 28 This assay uses human aquaporin-4 protein as an antigen, using M1 or M23 isoforms. Aquaporin-4 is transfected into live cells in research laboratories in large centers, but commercial kits also use prefixed transfected cells. Two reading techniques are routinely performed with similar specificities (immunofluorescence or flow cytometry). 22

The IPND-2015 recommends testing AQP4-IgG by CBA and repeating the exam if the result is negative in highly suspected patients. 1 Antibody testing is essential for NMOSD diagnosis, but there is no evidence of serial antibody testing yielding any practical advantage at the clinical follow-up. 47

Cell-based assay is also the recommended method for MOG-IgG testing. Patients who share NMOSD phenotype should be tested for MOG-IgG if AQP4-IgG is negative. Double seroprevalence of AQP4-IgG and MOG-IgG is extremely rare. 17 Patients presenting with monophasic or recurrent ON, myelitis, brainstem syndrome, or encephalitic features, especially children, should also be tested for MOG-IgG. 22

Following all the clinic, radiologic, and laboratory workup, the NMOSD diagnosis can be established as shown in Table 1 . A typical clinical syndrome is needed if the patient tests positive for AQP4-IgG. If the test is negative, there is a need for more than one typical clinical syndrome, one of them being ON, extensive myelitis, or area postrema syndrome to fulfill the radiologic criteria. 1

Most patients present a second attack within 3 years of the inaugural symptom, and ∼ 80 to 90% of all patients have a recurrent disease course. 1 Risks factors for recurrence are AQP4-IgG positivity, black ethnicity, age at onset. 48 Neuromyelitis optica spectrum disorder with positive AQP4-IgG is considered a more aggressive disease with a high potential for sequelae. Compared with patients diagnosed with MS or MOGAD, patients with AQP4-IgG NMOSD present with more severe attacks, lower recovery from attacks, and higher EDSS. 22 The disease is also responsible for a high impact on quality of life, chronic pain, and economic burden. 48

## TREATMENT CONSIDERATIONS

### Acute phase treatment

Although a few studies focused on evaluating acute therapies in NMOSD attacks, early and aggressive treatment is recommended to reduce the risk of permanent disability. 48 The same treatment is recommended for children and adults experiencing an NMOSD attack, and the doses are specified below. Historically, the first-line therapy is high doses of intravenous methylprednisolone for 3 to 5 days (20 tp 30mg/kg/day, maximum 1 g/day) followed by oral steroids tapering for 1–6 months. Second-line therapy, mainly plasma exchange (PLEX) for 5–7 cycles, is used for patients without good response to steroids. Recently, studies in adults demonstrated a better outcome when PLEX is prescribed within 5 days of symptoms onset. 22 A retrospective analysis of children with different acquired demyelinating disorders, including NMOSD, proved PLEX is an effective and safe therapy. Although no therapeutic window was identified in this study, the authors recommend PLEX treatment to achieve functional improvement. 49 Eventually, intravenous immunoglobulin (IVIG) is also a possible therapy for patients without recovery with the aforementioned treatments, especially after PLEX. The recommended dose is 1 to 2 g/kg prescribed in 2 to 5 days. 4 22

### Chronic phase treatment

All NMOSD patients with confirmed diagnosis should start long-term immunosuppression due to the high risk of recurrence and attack-related sequelae, 2 but more studies are needed to determine the duration of the therapies. 22 However, differential diagnosis with MS is required as disease-modifying therapies for MS, such as interferons, fingolimod, and alemtuzumab, can worsen NMOSD outcomes and should not be prescribed for NMOSD patients. 1

Oral immunosuppressants (azathioprine and mycophenolate mofetil) and especially the intravenous anti-CD20 medication (rituximab) are effective to prevent clinical attacks in adult and pediatric patients with NMOSD, 50 51 52 as shown in Table 2 .

**Table 2:** Long-term immunosuppressive drugs for preventing attacks in pediatric NMOSD patients

Medications	Dose	Adverse effects	Precautions
**Azathioprine**	2–3mg/kg/day, oral Once a day	Myelotoxicity and hepatotoxicity	-Check vaccinations and infection risks (including tuberculosis) before initiation. Avoid live vaccines. -Laboratory control to check myelotoxicity and hepatotoxicity monthly for 3 months and then every 3–6 months. -Tapper oral steroids after 3–6 months. -Contraception.
**Mycophenolate mofetil**	10–12,5mg/kg/day, oral (750–3,000mg/day) Twice a day	Myelotoxicity	-Check vaccinations and infection risks (including tuberculosis) before initiation. Avoid live vaccines. -Laboratory control to check myelotoxicity and hepatotoxicity monthly for 3 months and then every 3–6 months. -Tapper oral steroids after 3 months. -Contraception.
**Rituximab**	If child < 40kg or < 12 years old: -First infusion: 375mg/m2/dose, 4 doses, weekly -Subsequent infusions: 375mg/m2/dose, 2 doses, biweekly If child ≥ 40kg or ≥ 12 years-old: -First infusion: 1 g/dose, 2 doses, weekly -Subsequent infusions: 1 g/dose Repeat every 6 months or according to B cell repopulation evidence	Infusion allergies Hypoimmunoglobulinemia Hepatitis B reactivation	-Check vaccinations (specially hepatitis B vaccine) and infection risks (including tuberculosis) before initiation. Avoid live vaccines. -Pretreatment prior to infusion with antihistamines, antipyretics and corticosteroids -CD19 control at 2,4,and 6 months. -Laboratory control with cell blood count, IgG, IgM, and IgA every 3 months. -Contraception.

Table 2 summarizes the most commonly prescribed drugs to prevent NMOSD attacks in pediatric patients.

Recently, international agencies approved three drugs for AQP4-IgG-positive NMOSD evaluated by four clinical trials. The first approved drug, eculizumab, is a C5 inhibitor responsible for a risk reduction of 94.2% of attacks. 18 The efficacy of satralizumab, an interleukin 6 receptor blocker, was evaluated by 2 clinical trials providing a risk reduction of attacks of 55 and 62% if prescribed on monotherapy or in addition to oral immunosuppressants, respectively. 19 20 The study of inebilizumab, an anti-CD19, demonstrated a risk reduction of attacks of 79%. 21 If drug availability is not an issue, there is a recommendation to prescribe these medications as first-line immunosuppression for adults. 22

Other medications less commonly used are methotrexate, tocilizumab, and chronic oral steroids. All these treatments were evaluated in studies suggesting some reduction of NMOSD attacks.

A few studies evaluated symptomatic treatments for NMOSD, especially in the pediatric population. Treatment for chronic pain and tonic spasms consists of anticonvulsants, antidepressants, and analgesic drugs. Muscle relaxants are used to treat spasticity and anticholinergics to treat neurogenic bladder. 22 53

### Pediatric NMOSD

In 1927, there was the first report of NMOSD in patients < 18 years old. It is possible that many cases described earlier were misdiagnosed as other demyelinating disorders, especially MS. 54

The International Pediatric Multiple Sclerosis Study Group (IPMSSG) published the pediatric diagnostic criteria for MS and related disorders in 2007 and revised them in 2013. 55 The IPMSSG-2013 criteria have similar information to the adult NMO criteria published in 2006. Although the criteria have not been updated since then, the group published in 2016 a statement through a series of papers, considering that the IPND-2015 diagnostic criteria should be applied for the pediatric population. 5 An American multicenter study in 2016 proved that the new criteria increased the sensibility of NMOSD diagnosis in children and adolescents. 56

More recently, studies comparing pediatric and adult patients demonstrated no difference in disease manifestation regarding clinical, radiologic, and laboratory features. 6 7 Nonetheless, the 2015 Panel highlights some caveats for the pediatric population. Longitudinally extensive transverse myelitis is less specific in children than in adults since it can be a feature of acute disseminated encephalomyelitis (ADEM) and pediatric MS. The cerebral syndrome is more frequent in pediatric patients than in adults. 1 The frequency of MOG-IgG in the pediatric population is higher than in adults. 17

Although there was an increase in the publications of pediatric-onset NMOSD, the recognition of the disorder is epidemiologically limited by the low incidence and prevalence. 7 Studies are generally retrospective, using different inclusion criteria, with high heterogeneity in antibodies assays, and they are scarce in countries with higher disease prevalence, 6 as described in Table 3 .

**Table 3:** Main studies in pediatric-onset neuromyelitis optica spectrum disorders involving at least five patients

Author, Year, Country	Diagnostic criteria	Number of patients	Age at onset, years ± SD (range)	Gender F:M	Non-white ethnicity	Recurrent course	ARR	AQP4-IgG (Assay)	MOG-IgG	Follow-up, years (range)	EDSS Last visit
**Camera, 2021** **United Kingdom**	2015	49	12 ± 4,1	7:1	61.2%	83.7%	0.31	100% (CBA)	NR	6.6 (0.2–33.4)	NR
**Paolilo, 2020** **Brazil | Europe**	2015	67	10.2 ± 3,6 (2–16)	4,1:1	56.7%	86.6%	1.05	100% (CBA)	0/67	4 (2–10)	2.0 (1–3.5)
**Lechner, 2020** **Germany|Austria**	2015	24	11 (3–17)	2.7:1	45.8%	NR	NR	67% (CBA)	16.7%	NR	NR
** Zhou, 2019 a** **China**	2015	33	6.8 (4,2–8,7)	1.5:1	NR	NR	NR	30% (NR)	55%	NR	NR
**Dahan, 2019** **Australia**	2015	5	12.2 (6–15)	1:1,5	40%	60%	NR	20% (CBA)	40%	3 (± 1.6)	1.0 (0–1)
**Zhou, 2019** **China**	2015 2006	31	14 (7–17)	4.2:1	NR	94%	0.73	74.1% (CBA)	9.7%	2 (0.3–14.5)	1.5 (0–7)
**Fragoso, 2019** **Brazil**	2015	27	1–17	3.8:1	48.1%	NR	NR	15/23 (65.2%) (IFI)	0/9	6 (1–34)	3.0 (0–10)
**Baghbanian, 2019** **Iran**	2015	10	13 (8–17)	4:1	NR	100%	NR	7 (CBA)	NR	NR	2.5
**Sepulveda, 2018** **Spain**	2015	5	−	1.5:1	NR	NR	NR	40% (CBA)	60%	NR	NR
**Boesen, 2018** **Denmark**	2015	5	12.2 (2.8–14.8)	1:4	NR	NR	NR	NR	NR	4.6 (0.3–9.4)	NR
**Fragomeni, 2018** **Brazil**	2015 2006	12	14 (5.1–17)	2.6:1	46.5%	NR	1.5 (± 1.8)	72.5% (IFI)	NR	6.7	4
**Hacohen, 2017** **United Kingdom**	2015	28	8 (5 -11)	2.5:1	53.5%	100%	NR	28.6% (CBA)	57.7%	4 (3–6.7)	1 (0–2)
**Yamaguchi, 2016** **Japan**	2006	10	9.5 (3 -15)	4:1	100%	90%	0.66	30% NR	NR	6.3	NR
**Chitnis, 2016** **United States of America**	2013 2015	38	10.2 (16m -17)	2.1:1	63.1%	NR	NR	65% NR	NR	2	2.25
**Lechner, 2016** **Germany | Austria**	2006	12	NR	NR	NR	NR	NR	25% NR	NR	NR	NR
**Absoud, 2015** **United Kingdom**	2006	20	10.5 (2.9 -16,8)	9:1	NR	90%	NR	60% NR	NR	6.1	NR
**Fragoso, 2014** **Brzsil**	2006	29	13 (5–17)	2.6:1	58.6%	NR	0.75	78% (IFI)	NR	NR	4.7 (± 2.7)
**Rostasy, 2012** **Europe**	2006	8	11 (± 6)	6.2:1	NR	87.5%	NR	25% (CBA)	38%	NR	NR
**Pena, 2011** **Venezuela**	2006 2007	6	5–13	5:1	100%	100%	0.75	80% NR	NR	NR	NR
**Collonges, 2010** **France**	2006	12	14.5 (4.1–17.9)	3:1	33.3%	NR	0.6	66.7% NR	NR	19.3	NR
**Huppke, 2010** **Germany**	2006	6	12 (3–17)	2:1	33.3%	50%		17% NR	NR	3	NR
**Banwell, 2008** **Canada / Argentina**	1999	17	10.4 (4.4–15.2)	3.2:1	31%	52.9%	NR	47% NR	NR	1.1 a 4.95	2.5 (0–8)
**Lotze, 2008** **United States of America**	2007	9	14 (1.9–16)	9:0	77.7%	100%	NR	78% NR	NR	4	NR
**McKeon, 2008** **United States of America**	2006	58	12 (4–18)	2.6:1	73%	93%	NR	100% (IFI)	NR	1	4

Abbreviations: ARR, annualized relapse rate; AQP4-IgG, antibody against aquaporin; EDSS, Expanded Disability Status Scale; F, female; M, male; MOG-IgG, myelin oligodendrocyte glycoprotein; NR, not reported.

Note: ^a^ just abstract available.

Table 3 summarizes the main studies of NMOSD in the pediatric population involving at least 5 patients. 52 56 57 58 59 60 61 62 63 64 65 66 67 68 69 70 71 72 73 74 75 76 77 78

In conclusion, pediatric NMOSD is a rare but important differential diagnosis in inflammatory CNS disease with onset during childhood. Aquaporin 4 antibody is critical for a definitive and precise diagnosis of NMOSD, while seronegative patients should be evaluated for MOG-IgG due to the high frequency of this antibody in pediatric cases. New treatments for NMOSD have been approved in the last few years, but only satralizumab included adolescents (not children) in the pivotal trials. Therefore, future studies with pediatric NMOSD are urgently required to provide safe and efficacious treatments for this group of patients.
